# Utility of right ventricular free wall systolic strain for prediction of systemic sclerosis associated pulmonary arterial hypertension

**DOI:** 10.1093/ehjci/jeaf181

**Published:** 2025-06-17

**Authors:** Briella K Egberts, Rebecca Perry, Wendy Stevens, David Prior, Richard Woodman, Sivabaskari Pasupathy, Susanna Proudman, Joseph B Selvanayagam

**Affiliations:** College of Medicine and Public Health, Flinders University, 1 Flinders Drive, Bedford Park, SA 5042, Australia; Department of Heart Health, South Australian Health and Medical Research Institute, Adelaide, Australia; Department of Cardiovascular Medicine, Flinders Medical Centre, Bedford Park, Australia; Department of Cardiovascular Medicine, Flinders Medical Centre, Bedford Park, Australia; Allied Health and Human Performance, University of South Australia, Adelaide, Australia; Department of Rheumatology, St Vincent’s Hospital, Melbourne, Australia; Department of Cardiology, St Vincent’s Hospital, Melbourne, Australia; College of Medicine and Public Health, Flinders University, 1 Flinders Drive, Bedford Park, SA 5042, Australia; College of Medicine and Public Health, Flinders University, 1 Flinders Drive, Bedford Park, SA 5042, Australia; Department of Heart Health, South Australian Health and Medical Research Institute, Adelaide, Australia; Faculty of Health Sciences, Adelaide University, Adelaide, Australia; Discipline of Medicine, University of Adelaide, Adelaide, Australia; Rheumatology Unit, Royal Adelaide Hospital, Adelaide, Australia; College of Medicine and Public Health, Flinders University, 1 Flinders Drive, Bedford Park, SA 5042, Australia; Department of Heart Health, South Australian Health and Medical Research Institute, Adelaide, Australia; Department of Cardiovascular Medicine, Flinders Medical Centre, Bedford Park, Australia

Systemic sclerosis (SSc) has the highest mortality among autoimmune connective tissue diseases, with a standardized mortality ratio of 4.0. Pulmonary arterial hypertension (PAH), affecting up to 12% of SSc patients, is the leading cause of death, with a 50% mortality rate within 3 years of diagnosis.^1^ Annual non-invasive screening for PAH in SSc patients enables earlier detection and initiation of disease-modifying therapies, thereby improving survival rates.^2,3^ Transthoracic echocardiography (TTE) estimates right ventricular systolic pressure to screen for PAH, however, this method can be unreliable when tricuspid regurgitation is insufficient for accurate measurement. Moreover, TTE may not detect RV dysfunction until advanced PAH stages, potentially missing early disease. To address these limitations, the Australian Scleroderma Interest Group (ASIG) developed an internationally recognized screening algorithm that integrates pulmonary function testing and NT-proBNP levels to enhance early detection of PAH in SSc patients.^1^ Although the ASIG algorithm demonstrates high sensitivity, its limited specificity may result in unnecessary right heart catheterisations (RHC).^1^ RV dysfunction is an established prognostic marker in PAH,^4^ yet accurate and reproducible assessment of RV systolic function remains challenging due to the chamber’s complex geometry. Speckle-tracking echocardiography offers an angle-independent method to quantify myocardial motion.^3^ RV free wall systolic strain (RVFWSS) has been shown to predict clinical deterioration and mortality in established PAH, independent of left ventricular parameters.^5^ Although prior studies have identified RVFWSS abnormalities in SSc patients without PAH, longitudinal data on RVFWSS changes preceding PAH onset remain limited. This study aimed to evaluate whether incorporating echocardiographic RVFWSS into the existing ASIG screening algorithm can provide incremental prognostic value in the early detection of PAH in patients with SSc.

This study involved retrospective analysis of prospectively collected data from participants enrolled in the Australian Scleroderma Cohort study across two Australian hospitals. All participants underwent routine TTE between 2007 and 2022. Ethics approval was granted by Central Adelaide Local Health Network Research Committee and the St Vincent’s Hospital Melbourne Research Committee.

We retrospectively analysed prospectively acquired TTE data from SSc patients undergoing routine PAH screening as well as healthy volunteers. Participants were categorized into four groups: (1) SSc patients who screened positive by the ASIG screening algorithm and had PAH consequently confirmed by RHC; (2) SSc patients with positive ASIG screens but subsequent negative RHC; (3) stable SSc patients with no RHC indication; and (4) healthy volunteers. All participants had no significant prior cardiac or respiratory disease. Echocardiographic analysis was performed using a RV focused apical 4 chamber view.

Ninety-seven participants (Group 1 = 39, Group 2 = 17, Group 3 = 26, Group 4 = 15) were studied. Overall, the groups were well matched for age, gender and disease subtype (*Table 1*). Abnormal RVFWSS (−20%)^3^ was detected in Group 1 at a mean of 29 ± 23 months before PAH diagnosis. In Group 2, the proportion of patients with abnormal RVFWSS increased from 18% at baseline to 35% at the final TTE, despite negative findings on RHC. The mean final RVFWSS in Group 2 was significantly lower than in Groups 3 and 4. In contrast, Groups 3 and 4 showed no abnormalities and had comparable RVFWSS values. All participants maintained normal left ventricular ejection fraction (LVEF), right ventricular fractional area change (RV FAC), and TAPSE at both baseline and final assessments. At baseline, peak tricuspid regurgitant (TR) velocity was normal across all groups. However, by the final TTE, elevated TR velocity was observed in both Groups 1 and 2. Incorporating RVFWSS into the ASIG screening algorithm increased the positive predictive value (PPV) for PAH diagnosis from 0.60 to 0.79 while maintaining a high negative predictive value, potentially reducing unnecessary RHC. Diagnostic accuracy was highest for the combined ASIG + RVFWSS model (AUC = 0.844) compared with ASIG alone (AUC = 0.771) or RVFWSS alone (AUC = 0.781). RVFWSS also showed excellent reproducibility, with an intraclass correlation coefficient of 0.93 for inter-observer and 0.95 for intra-observer measurements (both *P* < 0.0001). This retrospective study has some limitations, including missing echocardiographic data, and suboptimal image quality affecting 7.2% of scans.

**Table 1:** Baseline and follow up variables in PAH, SSc non-PAH and control groups

	Timepoint	Group 1 SSC-PAH (+ve ASIG +ve PAH) *n* = 39	Group 2 SSc (+ve ASIG −ve PAH) *n* = 17	Group 3 SSc (−ve ASIG) *n* = 26	Group 4- HV *n* = 15
Age (years)	Baseline	56 ± 12	59 ± 10	56 ± 11	49 ± 22
Females (%)	Baseline	94	92	88	86
NT-Pro BNP (ng/L) Median [IQR]	Baseline Final	132.9 [96.2–501.0] 197.0 [114.3–681.2]	140.0 [75.0–326.1] 291.0 [74.5–599.3]	122.0 [84.0–224.0] 169.0 [137.0–336.0]	N/A
TAPSE (cm)	Baseline Final	22.2 ± 0.2 22.6 ± 6.0	22.2 ± 0.3 20.3 ± 6.1	24.1 ± 0.4 23.9 ± 4.9	17 ± 0.3 18 ± 0.5
RV FAC (%)	Baseline Final	44.5 ± 7.9 40.0 ± 11.2	36.7 ± 14. 1 40.3 ± 7.8	42.5 ± 2.1 33.5 ± 7.1	52 ± 10.9 53 ± 11.2
Peak TR velocity (m/s)	Baseline Final	2.8 ± 0.3 3.3 ± 0.5	2.7 ± 0.3 2.9 ± 0.5*	2.5 ± 0.3 2.3 ± 0.3	2.1 ± 0.2 2.2 ± 0.2
LVEF (%)	Baseline Final	61.0 ± 5.3 62.4 ± 5.4	61.4 ± 7.9 59.4 ± 9.5	60.1 ± 5.3 65.5 ± 0.7	61.5 ± 4.9 61.1 ± 6.6
RV global strain (%)	Baseline Final	−19.6 ± 4.0 −16.7 ± 2.8#	−19.7 ± 4.0 −18.9 ± 3.8*	−21.9 ± 1.6 −22.4 ± 1.8	−20.7 ± 3.8 −20.2 ± 2.8
RV free wall systolic strain (%)	Baseline Final	−23.5 ± 5.4 −18.9 ± 3.9# *	−23.4 ± 4.1 −23.1 ± 4.7*	−26.5 ± 1.7 −27.12 ± 2.1	−25.0 ± 0.3.1 −24.0 ± 3.3
RV basal strain (%)	Baseline Final	−25.2 ± 5.4 −20.2 ± 6.2#	−26.3 ± 6.4 −24.8 ± 4.0*	−26.8 ± 5.9 −25.8 ± 7.0	−22.8 ± 4.9 −22.2 ± 5.6
RV mid strain (%)	Baseline Final	−22.9 ± 5.9 −18.0 ± 4.2#	−23.1 ± 4.0 −22.2 ± 3.9	−25.4 ± 3.1 −25.7 ± 2.3	−23.71 ± 3.7 −22.2 ± 5.6
RV distal strain (%)	Baseline Final	−21.5 ± 7.2 −18.6 ± 3.3#	−19.6 ± 5.1 −20.6 ± 6.6	−23.8 ± 1.2 −23.7 ± 1.8	−23.2 ± 4.3 −24.7.0 ± 3.5
LV-GLS (%)	Baseline Final	−18.5 ± 3.0# −16.7 ± 2.8#	−18.7 ± 3.5# −18.9 ± 3.8#	−23.7 ± 3.6 −22.4 ± 1.8	−19.1 ± 3.6 −21.2 ± 3.7
RVFWSS ≥ −19.8%, *n* (%)	Baseline Final	9 (23%) 36 (92%) #*	3 (18%) 6 (35%) #*	0 (0%) 0 (0%)	0 (0%) 0 (0%)

Data presented as mean ± standard deviation (SD), *n* (%) or otherwise indicated. #*P* < 0.05 compared with normal control group, *P < 0.05 c/w baseline value. An abnormal RVFWSS was defined as a value of ≥ −19.8%, representing 2 SD below the cut-off established from the baseline healthy volunteer group. This was also comparable to the EACVI/ASE guidelines cut-off of > −20%.^3^

LVEF, left ventricular ejection fraction; LV-GLS, left ventricular global longitudinal strain; RV FAC, right ventricular fractional area change; RV, right ventricle; RVFWSS, right ventricular free wall systolic strain; TAPSE, tricuspid annular plane systolic excursion; TR, tricuspid regurgitation.

Our study underscores the significant role of RVFWSS as an early biomarker for PAH in patients with SSc. We found that RVFWSS abnormalities can precede a formal PAH diagnosis by an average of 29 months, suggesting RV dysfunction emerges well before overt haemodynamic changes. This mirrors findings by Fine *et al*.^5^ who reported significantly impaired RVFWSS in PAH and a 1.5-fold increase in mortality risk for every ∼ 7% decline in RVFWSS. The incorporation of RVFWSS into existing screening algorithms, such as the ASIG, substantially improves diagnostic accuracy and PPV, thereby potentially reducing unnecessary RHCs and enabling earlier intervention for at-risk patients. Future longitudinal research is needed to validate RVFWSS as a reliable predictor of PAH in SSc and explore its long-term implications.

## Data Availability

The data that support the findings of this study are available from the corresponding author, J.B.S., upon reasonable request.
